# Vascular changes in the cycling and early pregnant uterus

**DOI:** 10.1172/jci.insight.163422

**Published:** 2023-06-08

**Authors:** Noura Massri, Rachel Loia, Jennifer L. Sones, Ripla Arora, Nataki C. Douglas

**Affiliations:** 1 Cell and Molecular Biology Graduate Program and; 2 Institute for Quantitative Health Science and Engineering, Michigan State University, East Lansing, Michigan, USA.; 3 School of Graduate Studies, Rutgers Biomedical and Health Sciences, Newark, New Jersey, USA.; 4 Veterinary Clinical Sciences, School of Veterinary Medicine, Louisiana State University, Baton Rouge, Louisiana, USA.; 5 Department of Obstetrics, Gynecology and Reproductive Biology, Michigan State University, East Lansing, Michigan, USA.; 6 Department of Obstetrics, Gynecology and Reproductive Health and; 7 Center for Immunity and Inflammation, Rutgers Biomedical and Health Sciences, Newark, New Jersey, USA.

## Abstract

Uterine vascular remodeling is intrinsic to the cycling and early pregnant endometrium. Maternal regulatory factors such as ovarian hormones, VEGF, angiopoietins, Notch, and uterine natural killer cells significantly mediate these vascular changes. In the absence of pregnancy, changes in uterine vessel morphology and function correlate with different stages of the human menstrual cycle. During early pregnancy, vascular remodeling in rodents and humans results in decreased uterine vascular resistance and increased vascular permeability necessary for pregnancy success. Aberrations in these adaptive vascular processes contribute to increased risk of infertility, abnormal fetal growth, and/or preeclampsia. This Review comprehensively summarizes uterine vascular remodeling in the human menstrual cycle, and in the peri- and post-implantation stages in rodent species (mice and rats).

## Introduction

Under physiologic conditions, most vessels in the body are highly stable once formed. Blood vessels in the mammalian uterus are an exception, with characteristic structural and functional changes in both the non-pregnant and pregnant states (1, 2). In humans, in the absence of pregnancy, ovarian-derived estrogen and progesterone mediate cyclic vascular remodeling in the endometrium (1–6). In early pregnancy in rodents and humans, uterine vascular remodeling supports embryo growth and development and subsequent placenta formation (7, 8). Abnormalities in the uterine vasculature increase the risk of infertility, miscarriage, and pregnancy complications such as fetal growth restriction and preeclampsia. Thus, the normal physiologic vascular changes that occur during pre-pregnancy and early pregnancy are likely evolutionarily selected to support pregnancy success. Due to ease of visualization and relationship to the placenta, most documented changes in uterine vasculature have extensively focused on post-placental stages of pregnancy, and a discussion of uterine vasculature prior to and in early stages of pregnancy is missing (7, 9). Here, we describe uterine vasculature anatomy in both humans and rodents and factors that regulate vascular remodeling during the menstrual cycle for humans and the pre-placentation stages of rodent pregnancy. Uterine spiral arterioles undergo clinically significant adaptations during rodent and human pregnancy and have been more extensively investigated than uterine veins; these vessels and their derivatives are the focus of this Review.

## Human uterus anatomy and the menstrual cycle

**Figure 1:**
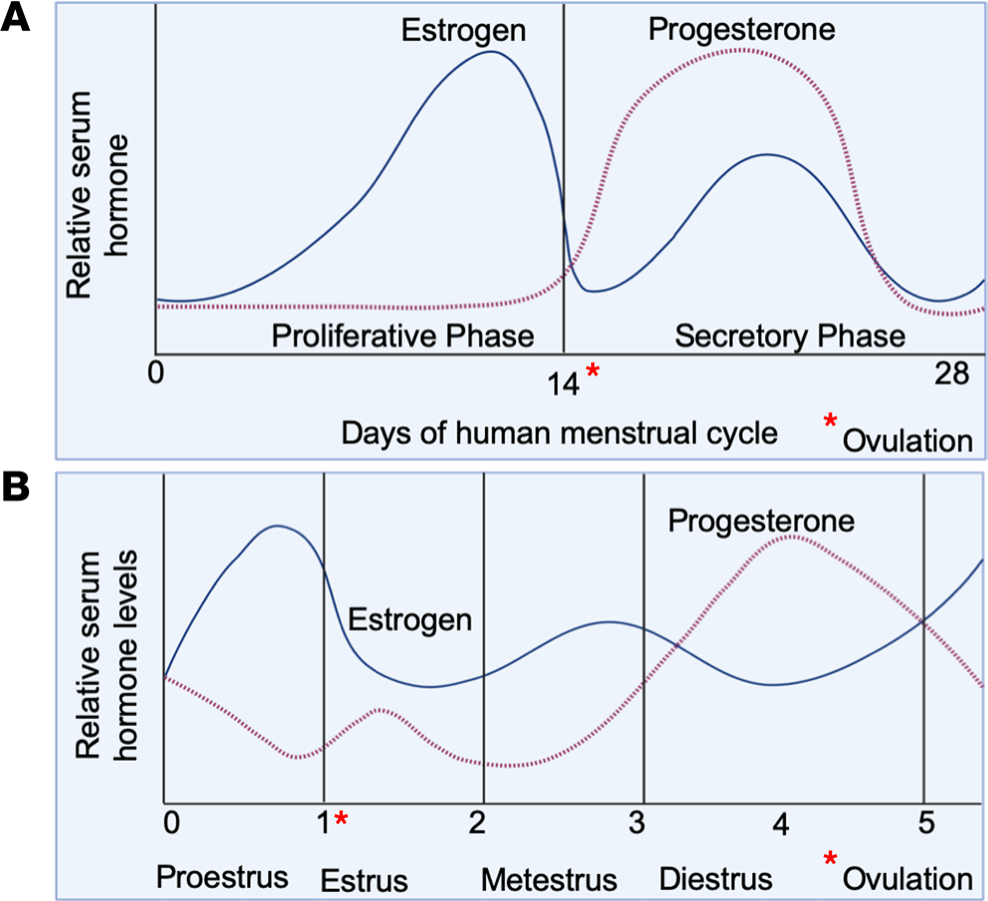
Comparison of the human menstrual cycle and rodent estrous cycle. (**A**) The human menstrual cycle is divided into the proliferative (follicular) phase and the secretory (luteal) phase. Under the influence of ovarian estrogen (blue line) in the follicular phase, the endometrium regenerates, endothelial cells proliferate, and vascular permeability increases. Mid-cycle, a surge in luteinizing hormone results in follicle rupture, ovulation of the oocyte, and formation of the corpus luteum. In the secretory phase, progesterone (red dashed line) regulates stromal cell decidualization and increases endometrial edema in preparation for embryo implantation. In the absence of embryo implantation, the corpus luteum demises, and estrogen and progesterone levels fall. This is followed by menstruation and the start of another menstrual cycle. (**B**) The rodent estrous cycle is composed of four different phases: proestrus, estrus, metestrus, and diestrus. In the proestrus phase, which lasts between 21 and 32 hours, estrogen increases. The estrus phase and ovulation follow the proestrus phase. The estrus phase lasts 12–48 hours. The formation of the corpus luteum following ovulation marks the shift from an estrogen- to a progesterone-dominated stage. The metestrus and diestrus phases represent the secretory phases of the cycle. The metestrus phase lasts 8–24 hours, while the diestrus phase lasts 48–72 hours.

The human uterus is composed of the myometrium, a smooth muscle layer; the endometrium, an inner mucosal lining surrounding mesenchymal stroma; immune cells; and an extensive vascular network (10). The endometrium is further divided into the deep stratum basalis, adjacent to the myometrium, and the superficial stratum functionalis. The normal human menstrual cycle lasts 21 to 35 days and is divided into the estrogen-dominated proliferative phase, which has marked endometrial layer thickening, and the progesterone-dominated secretory phase (Figure 1A) (10). Ovulation, with oocyte release and corpus luteum formation, marks the beginning of the secretory phase and progesterone-mediated endometrial differentiation in preparation for embryo implantation. In the absence of pregnancy, the endometrium and associated vasculature are shed in response to the demise of the ovarian corpus luteum and withdrawal of ovarian hormones. Thus, with each cycle, the functionalis layer responds to estrogens and progesterone, is shed with menstruation, and then regenerates from the basalis layer (10, 11). Endometrial hypoxia and vasoconstriction of spiral arterioles are thought to limit the amount of blood lost during menstruation (12).

## Types of blood vessels in the human endometrium

Uterine vasculature comprising both blood and lymphatic vessels has been consistently observed in the human myometrium (13). In contrast, in the endometrium, there are conflicting reports about the location of lymphatic vessels, while blood vessels have been well characterized (13–16). Blood vessels localize to three regions of the human endometrium: the subepithelial zone, functionalis layer, and basalis layer. The subepithelial zone extends to less than 200 μm below the surface epithelium, the basalis layer extends to less than 300 μm above the myometrium, and the functionalis is between (17). Uterine arteries are located within the myometrium and serve as the major blood supply to the uterus, branching into the arcuate and then the radial arteries. At the myometrial-endometrial junction, the radial arteries split to form basal arteries, which supply the basalis layer, and spiral arterioles, which supply the functionalis layer. Arteries in the basalis consist of endothelial cells associated with vascular smooth muscle cells (VSMCs) that express α-smooth muscle actin (α-SMA), γ-SMA, and myosin heavy chain. As the vessels course toward the luminal epithelium, VSMC content is reduced. Capillaries branching off from the spiral arterioles are present throughout the stroma and around the glandular epithelium. Beneath the luminal epithelial lining of the endometrium, capillaries arising from the spiral arterioles contribute to the subepithelial capillary plexus (3, 18). Microvessels at the subepithelial luminal surface are devoid of mural cells, including VSMCs and pericytes, and consist only of endothelial cells.

## Factors regulating the human endometrial vasculature

Cyclic changes in the endometrial vasculature in response to ovarian steroid hormones are associated with angiogenic factor activation.

### Steroid hormones

Changes in the endometrial vasculature during the menstrual cycle are thought to be directly regulated by estrogens and progesterone. Localized expression of estrogen and progesterone receptors in the functionalis layer has been described in endometrium from hysterectomy specimens. Estrogen receptor 1 (ESR1) and ESR2, previously described as estrogen receptors (ERs) α and β, respectively, and progesterone receptor (PR) are expressed in VSMCs (19). ESR1 is the predominant ESR during the late follicular phase, and ESR2 is predominant in the late secretory phase, suggesting hormonal regulation of their expression (19, 20). Whereas both ESR and PR are expressed in isolated human endometrial endothelial cells (21), endothelial cell expression of ESR2 has been detected in both proliferative- and secretory-phase endometrium, whereas PR expression has been variably detected (20–22). Thus, vascular remodeling could be mediated by acutely hormone-sensitive hormone receptor–expressing endothelial cells and VSMCs.

### Angiogenic factors

The endometrial expression of angiogenic factors, VEGF, angiopoietins (ANGPTs), FGF, and their receptors implicates involvement in regulating vascular changes in the proliferative and secretory phases (21, 23). VEGF is known to promote de novo blood vessel growth, angiogenesis, and vascular permeability via tyrosine kinase family receptors, including VEGFR1 (FLT1), VEGFR2 (KDR/FLK1), and VEGFR3 (FLT4) (24). In both the proliferative and secretory phases, VEGFA is expressed in neutrophils associated with the subepithelial capillary plexus and in microvessels in the functionalis layer (25). In the proliferative phase, VEGFA expression also localizes to glandular epithelial and stromal cells (23, 26), and VEGFR2 is found in endothelial cells (23). In the secretory phase, uterine natural killer (uNK) cells express high levels of the angiogenic factors VEGFC, placental growth factor (PGF), and VEGFR3. VEGFA expression is higher in the epithelium than in the stroma. In the secretory phase, endothelial VEGFR2 levels decline, whereas VEGFR1 levels increase (23, 26). In proliferative and secretory endometrium, ANGPT1 is predominantly expressed in stromal cells, while ANGPT2 and the angiopoietin receptor TEK receptor tyrosine kinase (TIE2) are highly expressed in endothelial cells (27). FGF1 and FGF2 are expressed in the epithelium and stroma, with higher expression in the epithelium throughout the cycle (23). Isolated human endometrial endothelial cells proliferate in response to VEGF or FGF2, and addition of 17β-estradiol increases, while progesterone decreases, endothelial cell proliferation in response to these angiogenic growth factors (21).

## Vascular remodeling in the human menstrual cycle

### Mechanisms of angiogenesis

Angiogenesis can occur via three mechanisms: sprouting, elongation, or intussusception, each requiring unique changes in endothelial cells (Table 1). Endothelial cell expression of α_v_β_3_ integrin, a marker of vascular sprouts in non-endometrial tissues (28), has been detected only inside existing vessels and not in any sprout-like vascular structures. This observation suggests that elongation and intussusception and not sprouting angiogenesis are the primary mechanisms of angiogenesis in the human endometrium (20). The following sections highlight the roles that VEGF signaling might play in the cyclic remodeling of the human endometrial vasculature.

### Angiogenesis during proliferative phase

In the proliferative phase, estradiol, the primary ovarian estrogen, promotes rapid endometrial growth and angiogenesis. Stereologic examination of vessel length and branch points showed that vascular length density in the subepithelial capillary plexus is greatest during the mid to late proliferative phase. These observations are consistent with vessel elongation as the major mechanism of endometrial angiogenesis in the estradiol-dominated proliferative phase (17). Fine spatial analysis revealed a greater percentage of proliferating endothelial cells in the basalis layer during the proliferative phase compared with the secretory phase. In contrast, the proportion of proliferating vessels in both the subepithelial capillary plexus and functionalis layer was relatively constant across the menstrual cycle (25). Estradiol increases endothelial cell proliferative response to VEGF and promotes elongation angiogenesis and vascular permeability (20). A significantly greater percentage of VEGF-expressing vessels and more proliferating vessels with VEGF-expressing cells were observed in proliferative compared with the secretory endometrium for all endothelial layers evaluated (subepithelial capillary plexus, functionalis, and basalis). VEGF displayed higher expression in the subepithelial capillary plexus compared with the functionalis and basalis layers. Finally, the observation of focal VEGF in marginating and adherent neutrophils associated with microvessels implicates neutrophils as the primary intravascular VEGF source for microvessels undergoing angiogenesis (25).

### Secretory-phase angiogenesis

Vascular development in the progesterone-dominated secretory phase is thought to occur via intussusceptive angiogenesis (3). Structurally, spiral arterioles increase in size and tortuosity, and the subepithelial capillaries dilate during the secretory phase (29). Microvessel concentration is thought to significantly increase in the mid-secretory phase (29). After ovulation, VEGFR2 levels decline, whereas VEGFR1 levels increase. While VEGFR2 signaling in the proliferative phase promotes endothelial cell proliferation, VEGFR1 signaling is thought to increase endothelial cell migration in the secretory phase (23). Unlike endothelial cells, mural cells proliferate under the control of progesterone during the secretory phase and wrap around endothelial cells, regulating angiogenesis and coordinating the function of blood vessels. In the late secretory phase, perivascular mural cells form a thick layer and surround the spiral arterioles (26). uNK cells increase in number during the mid-secretory phase and are located close to the endometrial glands and spiral arterioles, suggesting a role in the vascular remodeling that occurs in this phase (30).

### Vascular breakdown during menses

At the end of the secretory phase, right before menstruation, there is a rapid regression of the endometrium that induces increased coiling of spiral arterioles. Menstruation is associated with changes in the endometrial balance between matrix metalloproteinase (MMP) activators and tissue inhibitors of MMPs (TIMPs). Progesterone withdrawal seems to stimulate MMP activity, resulting in extracellular matrix (ECM) breakdown and bleeding. The transition from menses to the proliferative phase initiates with estradiol-independent repair of vascular beds during menstruation (29).

## The rodent estrous cycle

Like the human menstrual cycle, the rodent estrous cycle is orchestrated by ovarian-derived hormones that prepare the uterus for embryo implantation and pregnancy. In rodents, cyclic uterine remodeling occurs over 4–5 days (Figure 1B). Remodeling includes cell shape changes in the luminal and glandular epithelium and stromal cells, changes in uterine gland secretions, and changes in uterine lumen size and shape based on the volume of uterine luminal fluid (31). Although mice do not menstruate, there are mouse models of menstruation where endometrial shedding is induced after decidualization by withdrawal of progesterone (32). Despite these models, while a lot is known about the vascular remodeling events during the human menstrual cycle, little is known about vascular changes in the rodent estrous cycle.

## Types of blood vessels in the rodent uterus

**Figure 2:**
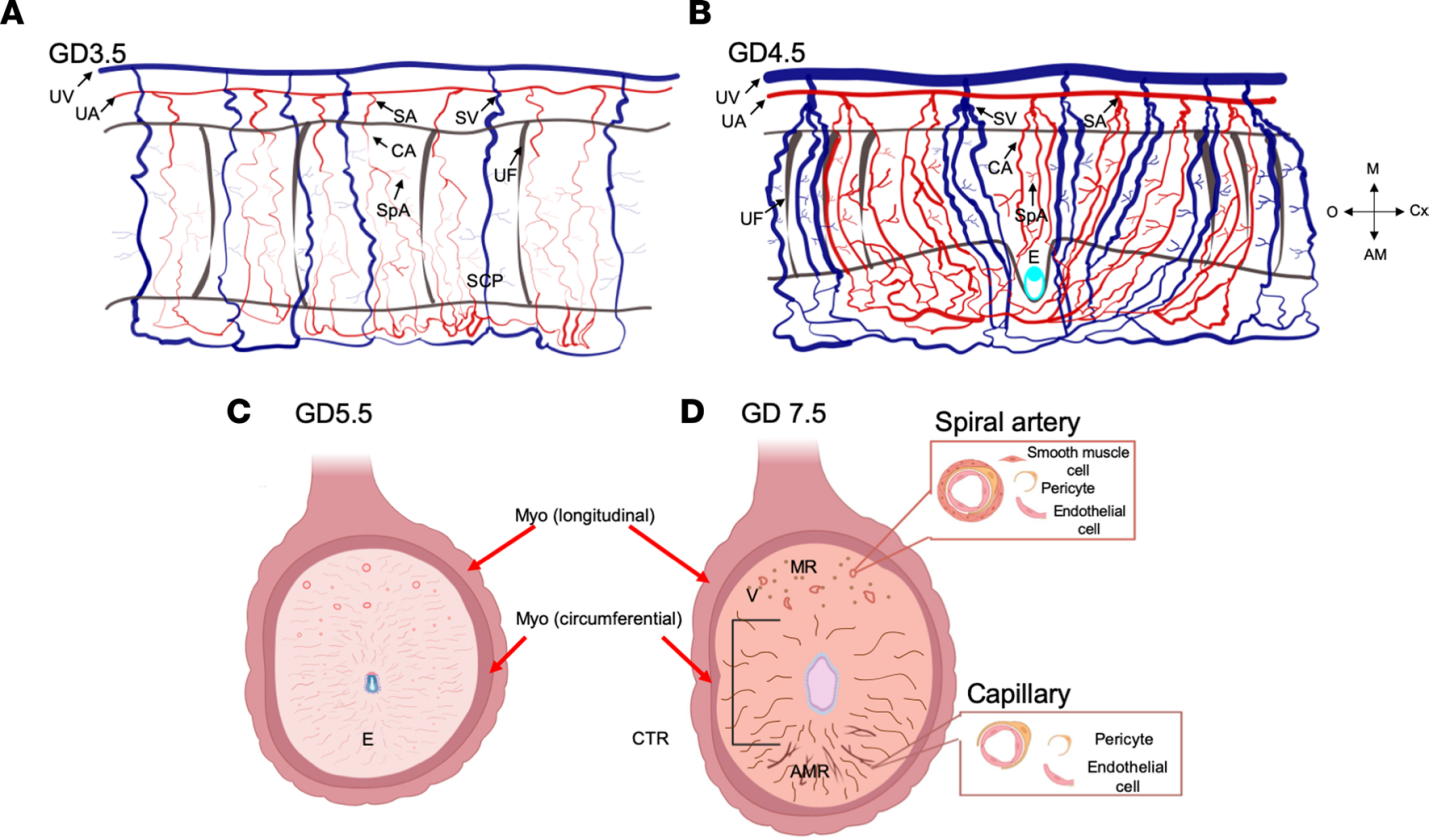
Vascular remodeling from pre-implantation through decidualization. (**A** and **B**) Longitudinal cross sections of the GD3.5 (**A**) and the GD4.5 (**B**) mouse uterus illustrating arteries (red), veins (blue), and the uterine lumen (outlined in gray). The utero-ovarian artery (UA) and vein (UV) give rise to the segmental arteries and veins, respectively (SA and SV). The segmental arteries divide into circumferential arterioles (CA) that then branch to form the spiral arterioles (SpA). Subepithelial capillary plexus (SCP) arise from the spiral arterioles and supply blood to the endometrium. Following embryo attachment at GD4.5 (**B**), the vessels remodel and dilate in proximity to the implantation region. (**C** and **D**) Transverse cross sections at GD5.5 (**C**) and GD7.5 (**D**). At GD5.5 (**C**) the newly formed decidual capillaries are readily apparent. By GD7.5 (**D**), the vasculature surrounding the implantation chamber can be divided into different regions — the central region surrounding the embryo (CTR), the mesometrial region (MR), and the anti–mesometrial region (AMR) — each with a unique composition of vessels. The MR is composed of spiral arterioles and capillaries. The spiral arterioles contain endothelial cells and mural cells (pericytes and VSMCs). The decidual capillaries in the AMR are smaller vessels composed of endothelial cells associated with pericytes, while the decidual capillaries in the CTR contain very few pericytes. AM, anti-mesometrial pole; Cx, cervix; E, embryo; M, mesometrial pole; Myo, myometrium; O, ovary; UF, uterine fold.

Like in humans, in rodents, both blood and lymphatic vessels have been consistently described in the uterine myometrium, while the endometrium and decidua of pregnancy appear to be devoid of lymphatic vessels (33). The rodent uterus is radial but asymmetric with large uterine arteries present along the mesometrial pole (Figure 2A). The uterine arteries, formed from the anastomoses of the uterine and ovarian arteries, are referred to as utero-ovarian or arcuate arteries. These arteries supply blood to segmental arteries in the mesometrium that branch into circumferential arterioles that pass through the uterine smooth muscle layers and wrap around the uterus. Circumferential arterioles further branch into spiral arterioles that supply blood to the inner muscle layers, the endometrium, and subepithelial capillaries present in proximity to the luminal epithelium (Figure 2A) (4).

## Events in early rodent pregnancy

Mating events in rodents are easily documented by detection of a copulation plug, termed gestational day (GD) 0.5. After mating, fertilization takes place in the oviduct (the rodent fallopian tube) followed by a series of cell divisions to form the blastocyst-stage embryo. In the mouse, multiple blastocysts enter the uterus early on GD3.0, followed by embryo spacing and embryo attachment on GD4.0 (34). Embryos attach to the uterine lumen, forming an implantation chamber (35), followed by a rise in vessel permeability around the site of attachment at GD4.5 (36). The implantation chamber then invades into the underlying stroma as the luminal epithelium around the embryo undergoes cell death and the stromal cells undergo a mesenchymal-to-epithelial differentiation process termed decidualization (36, 37). Between GD5.5 and GD7.5, the embryo continues to develop in the decidua, and embryonic umbilical vasculature fuses with maternal circulation by GD8.5 to form the placenta by GD9.5 (36, 37). Rat gestation is about 2 days longer than that of the mouse, and all events described above are shifted accordingly (38).

Limited information is available on early human pregnancy owing to lack of noninvasive methodologies to study the uterine microenvironment. Therefore, rodent models have been extensively used to examine endometrial changes required for successful embryo implantation and pregnancy progression. In the following sections, we describe several such rodent models and highlight human studies where applicable. Tables 2, 3, and 4 summarize additional factors not discussed in the text.

## Vascular remodeling in rodent pregnancy

### Vascular modifications in rodents during peri-implantation (GD0.5–GD4.5)

In rat pregnancy, endothelial cell proliferation is increased in the entire endometrium in the first few days following ovulation (39, 40). In rats and mice, as embryos enter the uterus, there is a substantial increase in the diameter of subepithelial capillaries in the endometrium (Figure 2B) (5, 41). Embryos attach to the anti-mesometrial pole and subepithelial capillaries in proximity to the implantation region dilate, leading to increased vessel permeability following embryo attachment (36, 42, 43). It is thought that the loss of vascular endothelial cadherin (VE-cadherin) facilitates this increase in permeability (44). In rodents, intravenous blue dye injection prior to euthanasia aids in the identification of embryo implantation sites owing to vascular leakage through permeable vessels (36, 43). Increased vascular permeability at implantation sites across several mammalian species, including sheep and pigs, underscores the importance of this vascular change for successful implantation and later pregnancy events (45–47).

### Factors contributing to increased vascular permeability (GD0.5–GD4.5)

During pre-implantation stages, the factors that contribute to increased endothelial cell proliferation and the exact timing of this process are not well described (39, 40). In contrast, increased vascular permeability around implantation sites has been heavily studied, and several factors have been implicated in this process (Table 2), as discussed below.

#### Steroid hormones.

In rodents, ovulatory estrogen induces epithelial proliferation at GD1–GD2, and a small rise in estrogen levels along with high levels of progesterone supports implantation by GD4.0 (48, 49). Experiments conducted in rodents to assess estrogen effects on the vasculature are typically performed with high estrogen levels, which mimic pre-ovulation conditions. Treatment of ovariectomized rats with estradiol or the estrogen agonist tamoxifen induced VEGF transcription (24, 50). Similarly, 17β-estradiol treatment of ovariectomized mice induced VEGF and VEGFR2 expression in the stroma within 6 hours of treatment (51). Further, a single injection of high-dose 17β-estradiol stimulated VEGF expression in the stroma within 2 hours after treatment (52). These studies imply a role for ovulatory estrogen in promoting expression of VEGF family genes. Like estrogen, progesterone alone induced stromal VEGFR2 expression in ovariectomized mice, although expression occurred within 12 hours of treatment. Thus, increase in serum progesterone levels may mediate the changes in uterine VEGF signaling that accompany embryo implantation (24, 51, 53, 54).

#### VEGF signaling.

The VEGF receptors, VEGFR1, VEGFR2, VEGFR3, and neuropilin 1 (NRP1) are expressed in the peri-implantation mouse uterus (24, 55), and endothelial VEGF signaling in the peri-implantation period is required for embryonic growth and pregnancy progression. VEGF164, the predominant VEGF isoform in the mouse uterus, is expressed in epithelial cells from GD0 to GD3 and in subepithelial stroma on GD2 and GD3. Following embryo implantation, VEGF164 is localized to the luminal epithelium and stromal cells surrounding the blastocyst (24). Interestingly, VEGF expression was elevated around embryo sites in early pregnancy but not during pseudopregnancy, suggesting that the presence of an embryo is necessary to induce expression between GD4.5 and GD6.5 (56). Systemic administration of a VEGF-blocking antibody in early rat pregnancy (GD2) lowered vascular permeability, reducing the number of implantation sites at GD4 without significant effect on microvascular density or endothelial cell proliferation (57). Thus, VEGF signaling plays a crucial role in pre-implantation vascular remodeling events associated with embryo attachment (57).

#### Prostaglandins.

Prostaglandin (PG) synthesis is necessary for the increased vascular permeability and uterine angiogenesis associated with early pregnancy. Prostaglandin synthase 2–derived (PTGS2-derived) PGs (PGE and PGI2) are elevated in implantation sites of several mammalian species (58–63). In rodents, depletion of PG function during pre-implantation is associated with abnormal post-implantation events, including implantation failure, decreased vascular permeability, and/or impaired decidualization, resulting in embryo growth restriction between GD5 and GD7 (45–47, 60, 62, 64–66). *Ptgs2^–/–^* mice also show defects in implantation and vascular permeability. However, daily intraperitoneal administration of the stable PGI2 analog carbaprostacyclin to *Ptgs2^–/–^* mice starting at GD3.75 improves implantation and restores VEGF and VEGFR2 expression, along with blood vessel numbers (61, 62, 65), suggesting that PG-depletion phenotypes are partly associated with disrupted VEGF signaling. Despite the association of both PG and VEGF signaling with increased vascular permeability in early pregnancy, the impact of PGs and VEGFs on vessel structure around the site of embryo attachment is not understood.

### Vascular modifications in rodents during decidualization (GD5.0–GD8.5)

From GD4.5 to GD7.5, decidual angiogenesis creates a rich, new capillary network (Figure 2C) (6). At GD5.5, the decidual transcriptome shows upregulation of endothelial cell–associated genes (67), with peak endothelial cell proliferation at GD6.5 (68). Thus, it is not surprising that differences in the decidual vasculature are readily apparent by GD7.5. The mesometrial region (MR) contains spiral arterioles covered with VSMCs and pericytes. The central region (CTR) contains capillaries with little or no pericyte coverage, while the capillaries in the anti-MR (AMR) are closely associated with pericytes (Figure 2D) (6, 69). Comparison of the GD7.5 vasculature to the estrous-stage non-pregnant vasculature revealed a 14.9-fold increase in the vascular sprouts in the CTR and a 7.4-fold increase in the vascular sprouts in the AMR. During early pregnancy, the intussusceptive blood vessels also increase 2.8-fold and 2.4-fold in the CTR and AMR, respectively. Thus, both sprouting and intussusceptive angiogenesis contribute to formation and remodeling of decidual vessels in the mouse.

### Factors contributing to increased vascular permeability and endothelial cell proliferation (GD5.0–GD8.5)

Post-implantation decidual angiogenesis and vascular remodeling, which includes the formation of new vascular networks, are regulated by key angiogenic signaling pathways (Table 3), including VEGF, ANGPT, and Notch (6, 67, 70, 71), and immune cells (72, 73), such as uNK cells (6, 74, 75), as discussed below.

#### Steroid hormones.

Similar to the pre-implantation/implantation stages, both estrogen and progesterone influence angiogenic factor expression in the post-implantation uterus. De novo synthesis of aromatase-derived estrogen from the stromal cells promotes angiogenesis during decidualization. Treatment with the aromatase inhibitor letrozole reduced expression of the endothelial cell–specific marker CD31 in the stromal compartment. Further, letrozole promoted downregulation of angiogenesis-related factors, such as endothelial PAS domain protein 1 (EPAS1), ANGPT2, and ANGPT4. These observations indicate that local estrogen production from the stromal cells induces secretion of angiogenic factors that contribute to endothelial cell network formation during decidualization (76).

Post-implantation treatment with a PR antagonist, RU486, decreased VEGF (6) and *Angpt2* expression at GD6.5 and GD8.5 and impaired uterine blood vessel remodeling at GD8.5 (70, 77). Spatiotemporal colocalization of PR and VEGFA was observed in decidual stromal cells at GD4.5. Further, while progesterone treatment at GD4.5 and GD5.5 increased blood vessel density in the CTR and AMR and increased vascular sinus fold numbers at GD6.5 in control mice, this progesterone-induced increase was not observed in VEGFA-deficient mice. Thus, progesterone-mediated changes in vascular remodeling primarily occur through VEGF and ANGPT2 signaling (6, 70).

#### Angiogenic factors.

After implantation, *Vegf* mRNA is expressed in decidual cells in the MR and AMR of implantation sites from GD5 to GD7 (24, 55). *Angpt2*, *Angpt3*, and *Tie2* are expressed in the endometrium starting after implantation at GD5 and reach maximum expression after decidualization at GD8.5 (61, 70). Another ANGPT family member, ANGPT4, is expressed in endometrial fibroblasts and endothelial cell populations during decidualization (78).

VEGF signaling via VEGFR1, VEGFR2, and VEGFR3 contributes to post-implantation vascular remodeling events (24, 55, 56, 68). Intraperitoneal administration of a VEGFR2-blocking antibody at GD3.75 reduced uterine vasculature at GD5.5, with subsequent embryo loss by GD10.5. VEGFR2 blockade in a pseudopregnant mouse model with oil stimulus also decreased uterine vasculature at GD6.5 (68), suggesting that a physical stimulus is sufficient to induce VEGF-dependent vascular changes. In contrast, although pre-conception and early pregnancy administration of VEGFR1- or VEGFR3-blocking antibodies reduced vascular density at GD5.5, embryo growth and pregnancy outcomes were not compromised. The phenotypes observed with VEGFA signaling blockade and conditional loss of one copy of *Vegfa* suggest that VEGF/VEGFR2 signaling promotes an increase in vascular density, enlargement of vascular sinus folds, and endothelial cell proliferation in the CTR and MR on GD4.5–GD8.5 (6). Together, these data suggest that VEGF/VEGFR2 is the only decidual VEGF signaling pathway essential for progression of pregnancy prior to placenta formation (6, 68, 79).

Studies in mice and women implicate aberrant VEGF expression in the decidua as the cause of later-stage pregnancy complications. The pregnant blood pressure high/5 (BPH/5) mouse is a superimposed preeclampsia model that exhibits placental defects, including shallow trophoblast invasion, inadequate spiral artery remodeling, and compromised fetal growth (80, 81). The decreased decidual vasculature observed in BPH/5 pregnancies at GD7.5 is associated with altered VEGF164, *Vegfr1*, *Vegfr2*, and *Plgf* expression (80). In the decidua of women with a history of recurrent pregnancy loss (RPL), *VEGF* mRNA and protein levels were significantly decreased in comparison with those without a history of RPL, who were undergoing voluntary termination of pregnancy (82). This observation suggests a link between disrupted VEGF signaling in early pregnancy and increased risk of miscarriage. Together, both mouse and human studies highlight the importance of VEGF signaling in decidual angiogenesis, placentation, and pregnancy success.

#### Notch signaling.

Like VEGF proteins, the distinct expression pattern of Notch proteins and ligands in the peri-implantation uterus reflects unique roles for Notch signaling in decidual angiogenesis and vascular remodeling (33, 69). Blockade of the Notch ligand delta-like ligand 4 (DLL4) at GD4.5 altered vascular tip and stalk cell identity, leading to excessive decidual angiogenesis and compromised embryonic growth by GD9.5 (71). Endothelial-specific deletion of the Notch regulator ADAM10 resulted in abnormal vascular patterning, impaired decidualization, and pregnancy loss by GD6.5 (67). In this model, there was increased diameter of blood vessels, a characteristic honeycomb structure of vessels near the implantation site, and increased endothelial cells in the AMR at GD5.5. In contrast, endothelial cell–specific loss of the Notch ligand Jagged1 at GD4.5 resulted in aberrant *Dll4* expression and increased Notch signaling without a change in the decidual vasculature or interruption of pregnancy at GD7.5 (69). These studies suggest a role for Notch signaling in decidual angiogenesis.

#### uNK cells.

uNK cells are immune cells that contribute to uterine vessel remodeling (83, 84). uNK cell precursors are present in the blood vessels and the stromal compartment in the pre-implantation mouse uterus (GD2–GD3). After implantation, uNK cells are abundant in the decidua and undergo notable maturation and proliferation from GD5.5 to GD7.5 (85). In addition, uNK cells produce soluble factors, including VEGF, PGF, and Notch ligand DLL1, that contribute to decidual angiogenesis and vascular remodeling during early pregnancy (73, 74, 85, 86). Between GD4.5 and GD6.5, uNK cells and VEGF/VEGFR3 signaling coordinately regulate enlargement and elongation of uterine vascular sinus folds in the MR (6). Even though uNK cells do not express estrogen and progesterone receptors in both mice (87–89) and humans (90), they are regulated by ovarian hormone signaling (72, 91). This is likely through steroid hormone regulation of stromal cells that surround the uNK cells (90). Consequently, ovarian stimulation (superovulation) with pregnant mare serum gonadotropin and human chorionic gonadotropin results in decreased uNK density near the MR of the decidua, deficient cytokine secretion, compromised placental angiogenesis, and low embryonic weight at GD8.5 (92).

Antibody-mediated depletion of uNK cells at GD6.5 and GD7.5 resulted in reduced blood vessel density and vascular sinus folding in the decidua CTR at GD8.5 (6, 75). There are conflicting data regarding the vascular phenotypes in uNK cell–deficient mouse models. IL-15 is a key cytokine for uNK cell maturation and is expressed in the decidual stromal cells (85). *Il15^−/−^* mice lack uNK cells and display expression of angiogenesis genes without abnormalities in vascular density or endothelial cell proliferation at GD7.5 (93). Conversely, a uNK cell–deficient model created by depletion of immune cells (*Rag2^–/–^ Il2rg^–/–^*) exhibits delayed mesometrial angiogenesis and maturation of decidual vessels at GD6.5, which compromises fetal development at GD9.5 (75). This study suggests involvement of uNK cells in promoting vascular branching and vessel pruning following implantation (75). However, in addition to uNK cell depletion, *Rag2^–/–^ Il2rg^–/–^* mice lack B and T cells (alymphoid). This may explain the differences in the two uNK cell–deficient models.

In humans, uNK cells are present in the endometrium and decidua of early pregnancy. These uNK cells express VEGFA and VEGFC, suggesting a role in decidual angiogenesis (26, 84). In vitro studies revealed that human uNK cells produce angiogenic factors (94) and that estrogen promotes uNK cell motility and uNK cell–mediated angiogenesis (95). After exposure to asoprisnil, a PR modulator, uNK cell numbers and IL-15 expression were significantly reduced in human secretory-phase endometrium. Impaired spiral artery remodeling, characterized by increased VMSC content and thickened arteriole walls, was also observed (96, 97). Increased numbers of uNK cells as well as increased vascular density and vascular wall thickness were observed in RPL compared with normal pregnancy (98).

#### Gap junction protein connexin 43.

Connexin 43 (*Cx43*), also known as Gap junction alpha-1 protein (Gjα1), is expressed in decidual stromal cells on GD4 and GD6. Mice with a dominant-negative loss of Cx43 function exhibited an increased and irregular pattern of decidual sinusoids at the mesometrial pole, decreased uNK cells at GD6.5 and GD7.5, upregulation of angiogenic factors such as VEGFA and FGF2, and fetal growth restriction (99). Global Cx43 loss results in postnatal death, preventing any analysis of fertility in the adult mouse (100). However, conditional deletion of *Cx43* in uterine stromal cells, epithelium, and the circular muscle via PR-Cre (*PR^Cre^*) reduced expression of ANGPT2 and ANGPT4 at GD6 and GD7. Uterine-specific Cx43-deficient mice also displayed reduced endothelial proliferation and a rudimentary vascular network on GD6 and GD7, resulting in poor decidualization and embryo development (101). These studies reveal the importance of intercellular communication via gap junctions in establishing the uterine vascular network that is necessary for decidual angiogenesis, embryo growth, and pregnancy success (82, 102, 103).

### Vascular remodeling in rodents during placentation (GD8.5–GD10.5)

Uterine vascular remodeling during placenta formation is mediated by decidual stromal cells, local uterine immune cells, including uNK cells, and embryo-derived trophoblast cells. When trophoblast cells in the trophectoderm layer of the blastocyst contact the endometrium, the mural trophectoderm differentiates into invasive trophoblast giant cells (TGCs), and the polar trophectoderm gives rise to the extraembryonic ectoderm and ectoplacental cone (EPC) (104). The EPC differentiates into several types of trophoblasts, including endovascular TGCs, interstitial glycogen trophoblast cells, and cytotrophoblasts, that serve unique functions in placental development in rodents (105). From GD8.5 to GD10.5, the major vascular changes are spiral arteriole remodeling and formation of the placenta.

#### Spiral arteriole remodeling.

**Figure 3:**
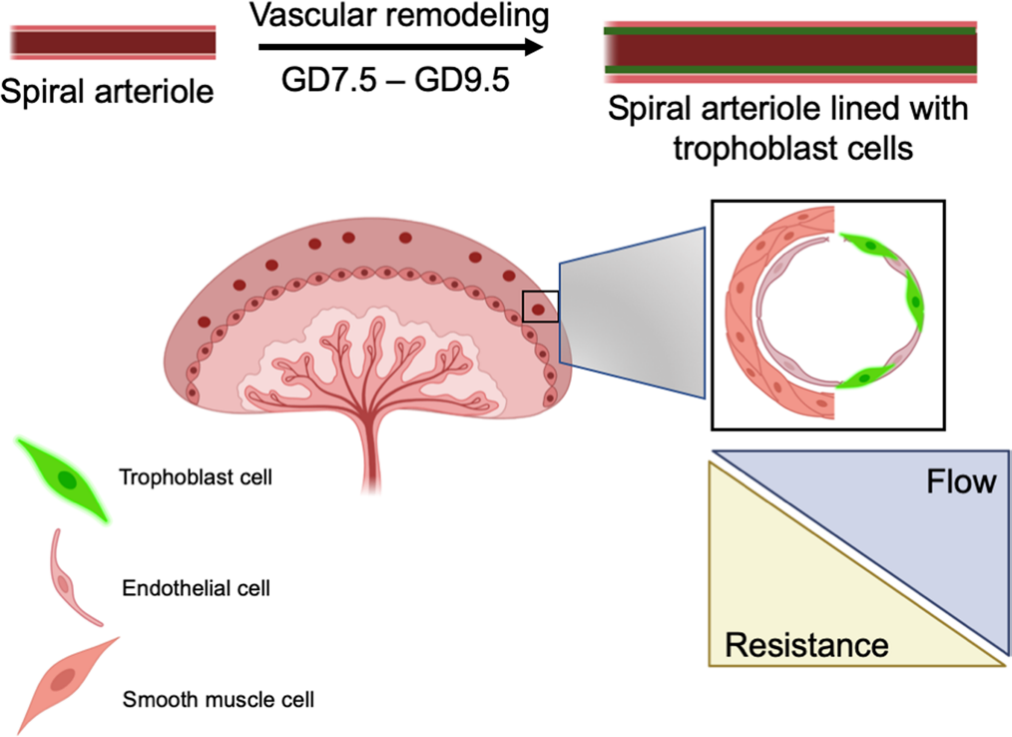
Remodeling of spiral arterioles during pregnancy. Spiral arterioles are lined by endothelial cells and are wrapped on the outside by pericytes and VSMCs. From GD7.5 to GD9.5, the maternal spiral arterioles increase in diameter after embryo-derived trophoblast cells (green) integrate into the lining of the vessel wall. As a result, the maternal vasculature in the pregnant uterus is one of decreased resistance and increased blood flow to nourish the fetus.

Spiral arteriole remodeling in rodents and women occurs via trophoblast-independent and trophoblast-dependent mechanisms. Trophoblast-independent, decidua-associated vascular remodeling involves endothelial vacuolation and VSMC swelling, leading to apoptosis. Trophoblast-dependent vascular remodeling involves the action of endovascular trophoblasts, the subtype of invasive trophoblasts that invades the arteriole wall, resulting in a chimeric vessel composed of both trophoblast and endothelial cells (Figure 3).

Interstitial and endovascular trophoblast invasion is required for placenta formation in rodents and women (106). In both species, interstitial trophoblasts degrade ECM proteins and invade the decidua to arrive at the spiral arteriole adventitial layer, whereas endovascular trophoblasts percolate through the vessel wall, restructure smooth muscle, and displace the endothelium (106). For rodent models of placentation, an important difference between rats and mice is the depth of trophoblast invasion. In rats, endovascular and interstitial trophoblasts invade beyond the decidua into the mesometrial triangle (or metrial gland) of the myometrium, while in mice, endovascular trophoblast invasion is limited, and interstitial trophoblasts do not invade into the myometrium (106). Furthermore, invasive trophoblasts in rats are intraluminal, replacing the endothelium from within the vessel, while those in mice are perivascular, replacing the endothelium from outside the vessel (106). Vascular changes in rodents and humans are similar. However, the depth of trophoblast invasion into the uterus and uterine vasculature and the intraluminal approach to vessel wall intercalation are most similar between rats and humans. Although the rat may be a more appropriate model of trophoblast invasion, the lack of transgenic rat models has limited investigations of molecular signaling underlying vascular changes. The mechanistic studies described below largely rely on mouse models, for which transgenics are more advanced.

Studies of spiral arteriole remodeling in mice use immunohistochemical staining of endothelial cells with vWF and VSMCs with SMA (107) to identify these key vasculature components. Remodeled spiral arteries have similarities to lymphatic vessels, including reduced SMA, lack of a basement membrane, and a large, dilated lumen (108). Spiral arterioles that were once high-resistance and low-capacitance are thus remodeled to low-resistance high-capacitance vessels that allow for adequate delivery of nutrients and oxygen from the mother to the developing fetus (Figure 3).

The placenta is fully formed by GD10.5 and is composed of three layers, the decidua, junctional zone, and labyrinth, each with unique vasculature. Within the maternal-derived decidua, arterioles undergo angiogenesis and trophoblast invasion after GD8.5 (73). The junctional zone, composed of spongiotrophoblast and glycogen trophoblast cells, is the confluence of maternal and fetal cells of the placenta. The labyrinth portion of the placenta is generally considered the fetal compartment, with fetal vascular endothelium and cytotrophoblast cells in direct contact with maternal blood (109). This type of placentation in rodents and humans, with intimacy of maternal and fetal cells for nutrient exchange, is classified as hemochorial (110).

### Non-trophoblast factors contributing to spiral arteriole remodeling (GD9.0–GD10.5)

Trophoblast-independent rodent spiral arteriole remodeling involves apoptosis and is mediated by angiogenic factors and uNK cells, as described below.

#### Apoptosis.

The process of endovascular and interstitial trophoblast invasion requires endothelial cell and VSMC apoptosis, as evidenced by the uterine expression of cleaved caspase-3 (111). Studies using mice deficient in uterine Corin, a serine transmembrane protease necessary for atrial natriuretic peptide (ANP) synthesis, showed that ANP is essential for decidualization and spiral arteriole remodeling (111). ANP-deficient mice demonstrated low expression of uterine TNF-related apoptosis-inducing ligand (TRAIL), which is required for VSMC apoptosis and cyclophilin B–mediated TRAIL receptor upregulation in endothelial cells within spiral arterioles (111). Gene mutations and reduced expression and activity of Corin have been associated with human preeclamptic pregnancy (112), suggesting that these mouse models could help develop a better understanding of the process of spiral arteriole remodeling.

#### uNK cells.

uNK cells are the predominant CD45^+^ immune cells in the mouse decidua until GD7.5 (73, 113, 114). Following IL-15 stimulation, uNK cells begin secreting key angiogenic and vasoactive factors, including VEGF, PGF, and IFN-γ, that are required for vascular remodeling in pregnancy. Retention of decidual vessel SMA in uNK cell–deficient *Rag2^–/–^ IL2rg^–/–^* mice results in constricted spiral arterioles at GD9.5 (115). At mid-gestation, mature uNK cells are essential for decidual maintenance. uNK cell–deficient mice have hypocellular decidual basalis at GD9.5–GD10 that becomes hypogranular at GD12 with spiral arterioles resembling those in non-pregnant mice (116).

#### Angiogenic factors.

A balance of pro- and anti-angiogenic factors in the placenta and maternal circulation is required for pregnancy success (Table 4). VEGF interacts directly with maternal uterine endothelial cells to promote angiogenesis and uteroplacental blood flow necessary for fetal circulation by GD9.5. Soluble VEGFR1 (sFlt1) is produced with pathologic placental hypoxia and scavenges free VEGF, preventing adequate placental angiogenesis (117). Paradoxically, VEGF overexpression in the endometrium of mice results in parallel upregulation of anti-angiogenic sFlt1 (118). Ultimately, endometrial VEGF overexpression in early pregnancy results in pathologically enlarged maternal blood spaces within the junctional zone while sparing labyrinth formation before GD11.5 (118).

Although early pregnancy studies are lacking in rats, a transgenic rat model of human preeclampsia was created by breeding of female rats containing a human angiotensinogen transgene with male rats harboring a human renin transgene (119). Uterine arterioles displaying a lower stretch response at GD7.5 correlated with a hypertensive pregnancy resembling the human preeclampsia condition.

### Trophoblast factors contributing to spiral arteriole remodeling (GD9.0–GD10.5)

Endovascular trophoblast cells replace endothelial cells of the spiral arterioles, transforming them from small resistant vessels to flaccid, large-caliber vessels that are unresponsive to vasoconstrictive agents. This complex process is thought to occur in rodents and women. At the inception, endovascular trophoblast cells form plugs in maternal uterine vessels. This is thought to protect the conceptus from excessively high oxygen levels, and thus placenta formation begins in a hypoxic state (73, 120). Once the plugs dissociate, maternal blood flow to the placenta increases. Notch is involved in trophoblast-dependent spiral arteriole remodeling prior to GD10.5.

#### Notch signaling.

Canonical Notch signaling is active in trophoblasts during early pregnancy, and trophoblast subtypes express Notch proteins, including DLL4, Notch2, and Notch4 (121). *Notch2* deletion using the trophoblast-specific driver *Tpbpa*-Cre resulted in inadequate remodeling of spiral arterioles and a decline in placental perfusion at GD10.5 and GD14.5 (122).

## Eye to the future

There are clear gaps in our understanding of uterine vascular remodeling during early pregnancy. Most studies describe vascular structure, gene expression, and, to some extent, functional changes in rodents (3, 24, 39–42, 57, 123). Very few studies simultaneously investigate signaling pathways and vessel architecture (6, 70). Future studies that combine genetic rodent models and advanced methodologies, such as organoids, organ-on-chip, and omics, with novel imaging techniques will allow integration of gene expression with spatial information and vessel function at different stages of the estrous/menstrual cycle and in early pregnancy.

Endometrial organoids and organ-on-chip represent promising systems that can facilitate study of tissue function in physiologic and pathophysiologic contexts. These systems may provide a suitable approach to bridge the gap between 2D cultures and in vivo models (124). Endometrial organoids have been generated using epithelial cells alone or epithelial and stromal cells together but have not yet integrated endothelial cells (125). An implantation-on-chip model has also been constructed to understand the interaction of primary trophoblast cells, which were derived from first-trimester placenta, with uterine stromal and uNK cells. In this model, trophoblast invasion and migration directionality were drastically reduced in the absence of uterine endothelial cells. Further, non-uterine sources of endothelial cells promoted trophoblast invasion to a much lesser extent compared with uterine endothelial cells, supporting the unique properties of organ-specific endothelium (77).

Transcriptomic profiling of the uterine microenvironment with RNA sequencing during different stages of pregnancy is emerging as a powerful tool to determine the expression patterns of angiogenesis genes that are necessary for implantation and pregnancy success (126–128). Single-cell RNA sequencing has the advantage of interrogating the transcriptome of specific cells within the heterogeneous placental tissue composed of maternal- and fetal-derived cells. Information on the cell-to-cell crosstalk between maternal and fetal cells can be investigated more elegantly with spatial transcriptomics (126). Equally important are new imaging advances, such as confocal and light sheet microscopy, that will allow connections between gene expression changes and vascular structural and functional changes during implantation, decidualization, and early pregnancy (125, 129).

## Conclusions

While a lot has been uncovered about uterine vascular changes during early pregnancy, many unanswered questions remain. The principal molecular signals in uterine (maternal) vascular remodeling during early pregnancy and how vessels are modified during clinical conditions such as infertility and miscarriage are not known. The downstream vasoactive and angiogenic pathways directly regulated by ovarian steroid hormones that result in vessel remodeling during the estrous cycle and during pre-implantation stages of pregnancy have not been well studied. Finally, although implied by increased vascular permability during early pregnancy, how the changes in blood vessel structure relate to function needs further attention. Addressing these gaps in knowledge will provide novel perspectives regarding early pregnancy and allow design of therapeutics to address clinical conditions related to infertility and miscarriage.

## Figures and Tables

**Table 1:**
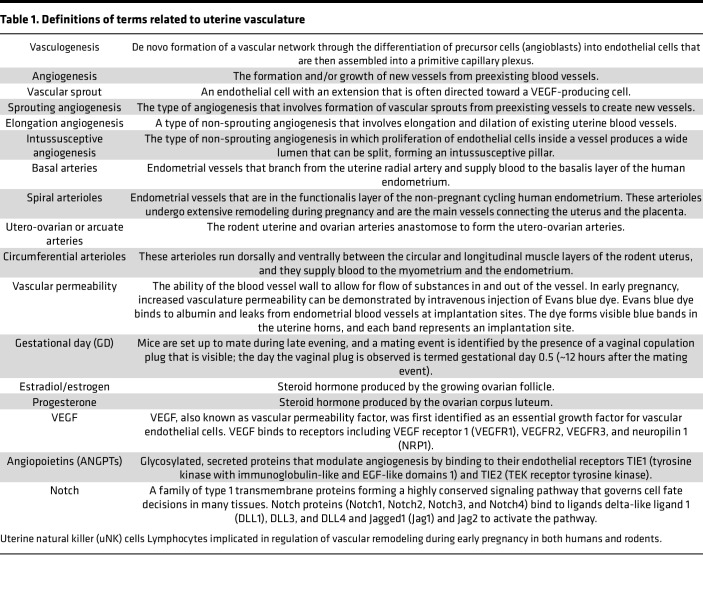
Definitions of terms related to uterine vasculature

**Table 2:**
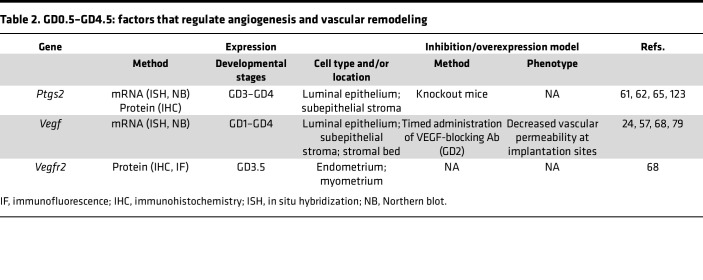
GD0.5–GD4.5: factors that regulate angiogenesis and vascular remodeling

**Table 3:**
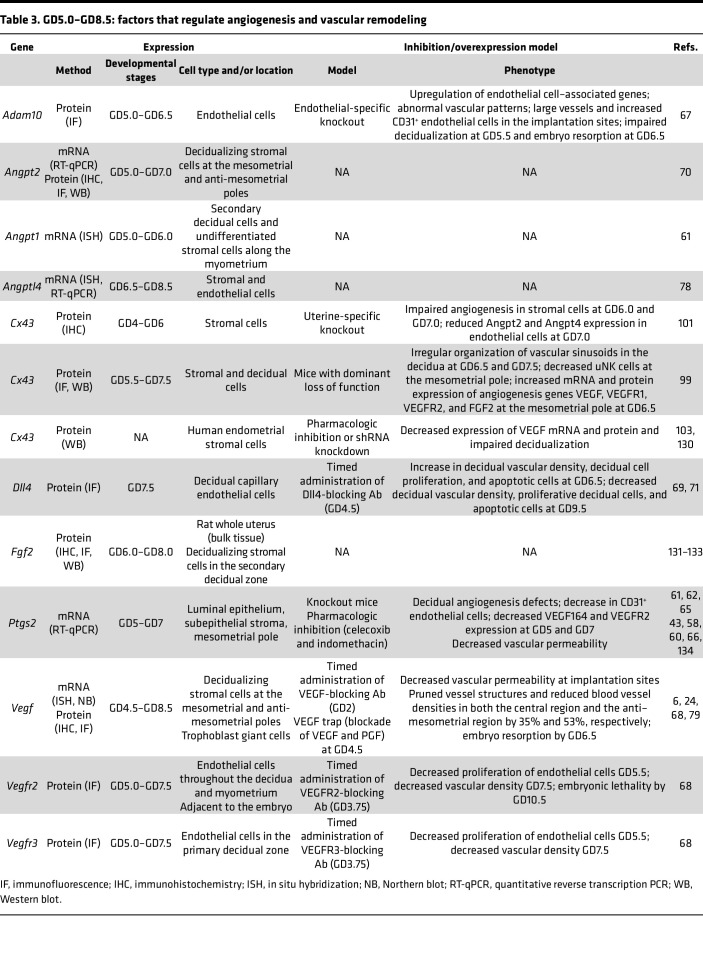
GD5.0–GD8.5: factors that regulate angiogenesis and vascular remodeling

**Table 4:**
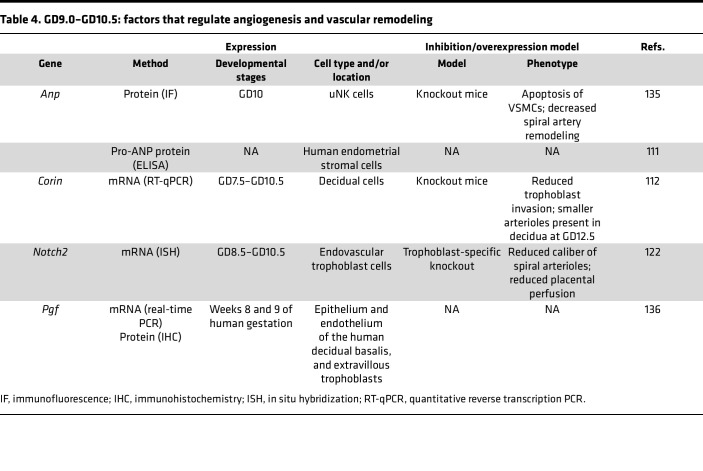
GD9.0–GD10.5: factors that regulate angiogenesis and vascular remodeling

## Version 1 06/08/2023

Electronic publication
